# Features of the Chemical Composition and Structure of Birch Phloem Dioxane Lignin: A Comprehensive Study

**DOI:** 10.3390/polym14050964

**Published:** 2022-02-28

**Authors:** Anna V. Faleva, Ilya I. Pikovskoi, Sergey A. Pokryshkin, Dmitry G. Chukhchin, Dmitry S. Kosyakov

**Affiliations:** Laboratory of Natural Compounds Chemistry and Bioanalytics, Core Facility Center “Arktika”, M.V. Lomonosov Northern (Arctic) Federal University, Northern Dvina Emb. 17, 163002 Arkhangelsk, Russia; i.pikovskoj@narfu.ru (I.I.P.); s.pokryshkin@narfu.ru (S.A.P.); d.chuhchin@narfu.ru (D.G.C.)

**Keywords:** birch phloem, lignin, nuclear magnetic resonance, high-resolution mass spectrometry, Py-GC/MS

## Abstract

Understanding the chemical structure of lignin in the plant phloem contributes to the systematics of lignins of various biological origins, as well as the development of plant biomass valorization. In this study, the structure of the lignin from birch phloem has been characterized using the combination of three analytical techniques, including 2D NMR, Py-GC/MS, and APPI-Orbitrap-HRMS. Due to the specifics of the phloem chemical composition, two lignin preparations were analyzed: a sample obtained as dioxane lignin (DL) by the Pepper’s method and DL obtained after preliminary alkaline hydrolysis of the phloem. The obtained results demonstrated that birch phloem lignin possesses a guaiacyl–syringyl (G-S) nature with a unit ratio of (S/G) 0.7–0.9 and a higher degree of condensation compared to xylem lignin. It was indicated that its macromolecules are constructed from β-aryl ethers followed by phenylcoumaran and resinol structures as well as terminal groups in the form of cinnamic aldehyde and dihydroconiferyl alcohol. The presence of fatty acids and flavonoids removed during alkaline treatment was established. Tandem mass spectrometry made it possible to demonstrate that the polyphenolic components are impurities and are not incorporated into the structure of lignin macromolecules. An important component of phloem lignin is lignin–carbohydrate complexes incorporating xylopyranose moieties.

## 1. Introduction

With the depletion of fossil fuel reserves, lignin is becoming increasingly important as the potential renewable source of aromatic substances, being the second most abundant (after cellulose) plant biopolymer in nature. It is well known that lignin macromolecules consist of phenylpropane units of three types, the *p*-hydroxyphenyl (H), guaiacyl (G), and syringyl (S), interconnected by various types of bonds [1]. Although the relative amount of each bond type depends on a number of factors, the key structures are common to all lignins and are represented by the β-aryl ether (β-O-4), phenylcoumaran (β-5 and α-O-4), resinol (β–β and α-O-γ), as well as biphenyl links (5-5 bonds) [2,3]. However, despite the long history of research, the exact structure of lignin is still unidentified. Studies on the chemical structure of lignin preparations isolated from various plants are ongoing, significantly expanding our knowledge about the biopolymer and its transformations in different processes.

The overwhelming majority of studies of woody plant lignins available in the literature were performed on preparations isolated from xylem. At the same time, bark lignins still remain poorly studied although the bark is a multi-tonnage waste of the pulp and paper and woodworking industries [4,5]. Among them, inner bark (phloem) lignins are of the greatest interest since phloem, unlike xylem, is a living tissue, the study of which allows for a new knowledge to be gained about the processes of lignin biosynthesis and the ontogenesis of anatomical elements of plant biomass. However, the works on obtaining the reliable data on the chemical composition and structure of the phloem lignin is only starting.

Previous papers [6,7,8] have reported the high lignin content in the phloem ranging from 18% to 38%. Two of them [7,8] are dedicated to oak and willow phloem lignins, respectively, and can be considered as the first works aimed at a detailed study of their chemical structure. Both independent studies have shown that phloem lignins differ significantly from cork and xylem lignins. Another most important object of research is birch phloem since birch bark is a multi-tonnage industrial waste and has been repeatedly considered as a source of valuable chemicals, including betulin [9] and suberin [10]. However, publications aimed at studying the structure of birch phloem lignin are not available in the literature except for in work [11], dealing with the products of lignin chemical degradation (CuO oxidation and thioacidolysis).

In the recent work of [12], it was found that the functional group composition of the birch phloem lignin had no significant differences from xylem lignins. However, the used analytical tools did not allow for proper assessment of its structural features. Moreover, the unusual pink color and a characteristic absorption maximum at 550 nm in the UV spectrum indicated the presence of polyphenolic impurities in the studied lignin sample. The latter can be condensed tannins and high-molecular polyphenolic acids (HPA), which are extracted from plant tissues along with lignin and can be effectively removed with only an alkali solution [13]. Polyphenols can participate in the formation of phloem lignin macromolecules due to covalent binding during lignin biosynthesis by analogy with cinnamic acids, which play an important role in the structure of grass lignins [14,15]. Another issue is the possibility of condensation of HPA with lignin to form new covalent bonds during a lignin isolation procedure [16]. This can significantly affect the results of lignin analysis and the identification of truly new lignin fragments. Therefore, it is necessary to conduct additional studies to establish the chemical composition and structure of phloem lignin obtained with a preliminary stage of alkaline hydrolysis.

Given the exceptional complexity of the chemical composition of phloem lignin preparations, in addition to common degradation methods (e.g., analytical pyrolysis successfully replacing traditional wet chemistry methods [11]), the most advanced analytical techniques should be used for their characterization. These include, primarily, two-dimensional (2D) NMR spectroscopy and high-resolution mass spectrometry. The first one provides information regarding the different lignin units and inter-unit linkages and is considered now as a most powerful tool for lignin structural studies [17]. Typically, it involves the most popular HSQC (Heteronuclear Single Quantum Coherence) experiments with recording of additional diagnostic 2D NMR spectra, for example, HMBC (Heteronuclear Multiple Bond Correlation) techniques to identify unknown cross-peaks [4,5,7,8,14,15,16,17,18]. An additional way to increase the reliability of identification is by using expert systems for the prediction of spectra and computer-assisted structure elucidation [18].

High-resolution mass spectrometry (HRMS) provides additional valuable information regarding the chemical composition of lignin at molecular level, which is complementary to 2D NMR data. One of the most promising analytical tools for molecular characterization of lignin is an orbital ion trap (Orbitrap) HRMS with atmospheric pressure photoionization (APPI) demonstrating a high efficiency of ionization of biopolymers in the wide range of molecular weights (up to 2 kDa) and a capability to simultaneously detect several thousands of oligomers [19,20]. This technique has been successfully used to characterize extremely complex mixtures of lignin depolymerization products [21,22], as well as to study the structure of nettle and sedge grass lignins [15,23].

A combination of 2D NMR spectroscopy, APPI-Orbitrap HRMS and pyrolytic gas chromatography–mass spectrometry (Py-GC-MS) was used in the present work for the maximum complete characterization of the poorly studied birch phloem lignin aimed at revealing the features of its chemical structure and role of polyphenolic substances in its formation.

## 2. Materials and Methods

### 2.1. Plant Material

The bark and wood of downy birch (*Betula pubescens*) collected in the spring of 2017 in the forest area of the Obozersky forestry in the Arkhangelsk region (Russia) were used as plant materials for isolation of birch phloem (BPL) and birch xylem (BXL) lignins. The average age of the trees selected for sampling was 50–60 years. Phloem was manually separated from the outer bark, finely ground in a ball mill (Retsch, Haan, Germany) and then subjected to sequential Soxhlet extraction with cold water (24 h), hot water (3 h), toluene (12 h) and acetone (12 h). The same milling and extraction procedures were used for xylem sample preparation.

After air drying, the obtained extractives-free phloem sample was divided into two parts, one of which underwent alkaline hydrolysis according to a known procedure [24]. Briefly, a weighed portion (2 g) of phloem powder was placed in a 250 mL conical flask and poured with 100 mL of 1% aqueous sodium hydroxide solution. After thorough mixing, the flask was heated on a water bath for 1 h with periodical stirring. After filtration, the precipitate was washed on a filter with hot distilled water, then with 10% acetic acid solution, and again with hot water. Further, the residue was vacuum dried to constant weight.

The mass fraction of substances soluble in a 1% sodium hydroxide solution was recalculated to the initial phloem sample (before Soxhlet extraction). Klason lignin (an insoluble residue after digestion with 72% sulfuric acid at 30 °C, then with dilute sulfuric acid at 100 °C to hydrolyze and solubilize the polysaccharides) content was determined according to the standard method described in [24]. The measured contents of lignin and extractives are presented in Table 1.

### 2.2. SEM Study

SEM microphotographs were obtained using Zeiss Sigma VP (Carl Zeiss, Oberkochen, Germany). The phloem tissue samples were prepared by cryo-mechanical destruction in liquid nitrogen (−196 °C) with further freeze drying. To increase the contrast of the images, a gold-palladium coating was sputtered to the surface of the samples using a Q150 TES coater (Quorum Tech., Laughton, UK).

### 2.3. Isolation of Dioxane Lignin

A main challenge in elucidating the structure of lignins is obtaining a high-yield sample in a chemically unaltered form. The organosolv dioxane lignin (DL) isolated from plant material by mild hydrolytic treatment and extraction with 1,4-dioxane is considered to be one of the most representative preparations of the native lignin [16], except for partial destruction of ether bonds and the formation of new condensed structures under the action of a hydrochloric acid catalyst [26].

Three DL preparations were isolated from birch phloem (DL-P), phloem after alkaline hydrolysis (DL-P-AH) and xylem (DL-X) by the common Pepper method [27]. Briefly, the phloem and xylem samples were treated by a dioxane–water (9:1 *v*/*v*) solution in the presence of hydrochloric acid (0.7%, *w*/*w*) in a water bath at 96 °C for 2 and 4 h in an argon atmosphere. The extraction time for the phloem sample was reduced as compared to xylem to avoid unwanted condensation of tannins in acidic medium. The obtained extracts were neutralized with sodium bicarbonate, filtered, and then concentrated on a rotary evaporator under reduced pressure at 25 °C. The lignin was precipitated by adding tenfold excess of water. After centrifugation, the lignin preparations were dried to constant weight under vacuum at 40 °C. To remove low-molecular weight impurities, lignin samples were precipitated from a dioxane solution to diethyl ether, and then air dried and kept under vacuum overnight to completely remove the residual solvents. The yields of the obtained DL-P, DL-P-AH and DL-X lignin preparations recalculated to Klason lignin were 30.3%, 24.7% and 25.0%, respectively. Weight-average (M_w_), number-average (M_n_) molecular weights and polydispersity index (PDI) determined by size-exclusion chromatography [15], as well as hydroxyl and carboxyl group contents determined by the conventional wet chemistry methods [28] for the obtained lignin preparations are presented in Table 2.

### 2.4. NMR Spectroscopy

The sample (~80 mg) of the isolated dioxane lignin was dissolved in 0.55 mL of DMSO-d6 (99.9%, Deutero GMBH, Kastellaun, Germany). All NMR spectra were recorded at 298 K on an AVANCE III 600 MHz spectrometer (Bruker Biospin, Rheinstetten, Germany). Specifically, 2D NMR spectra were recorded using an adiabatic HSQC pulse program (“hsqcetgpsisp2.3” and “hmbcetgpl3nd” standard pulse sequences). The [^1^H-^13^C] HSQC spectrum was registered with a delay equal to 2.0 s, the spectral width of 15 ppm in F2 and 238 ppm in F1 with 1024 × 256 increments, and 32 scans per increment. The [^1^H-^13^C] HMBC spectrum was registered with a delay equal to 2.0 s, the spectral width of 11 ppm in F2 and 240 ppm in F1 with 2048 × 512 increments, and 32 scans per increment. The solvent signal (DMSO-d_6_) was used as an internal standard (δC/δH 39.5/2.5 ppm). Then, the ^13^C NMR spectra were recorded using the “zgig30” sequence (from Bruker Standard Library), which allows for quantitative analysis and comparison of signal intensities. The following parameters were used: 12 ms pulse width; 1.4 s acquisition time; 2 s relaxation delay; 64 K data points; ~16,000 scans. The sample relaxation time was artificially reduced by the addition of chromium (III) acetylacetonate with a concentration of 5 mg mL^−1^.

Two-dimensional NMR HSQC cross-signals were assigned after comparison with data from the literature [29,30,31,32]. The correctness of the identification was confirmed by the presence of all characteristic cross-peaks of the indicated fragments and the compliance of their chemical shifts with the literature data. Comparative analysis showed that the cross-peak positions for the three studied lignin preparations differ from those published in the literature by <0.05 and <0.5 ppm in ^1^H and ^13^C dimensions, respectively (Table S1).

In case of detection of unknown peaks, their assignment was carried out by two approaches using ACD/Labs computational expert system (ACD/Labs, Toronto, ON, Canada). The first is based on the use of a database of NMR chemical shifts, taking into account the correlations present in the diagnostic NMR spectra by ACD/Structure Elucidator. The second approach suggests using ACD/Spectrus Processor and consists of two stages. The first stage involved constructing model spectra of the putative compounds using the HOSE-code algorithm. The accuracy of the calculation was determined based on the protocol for each atom in both elemental dimensions (Figure S1 and Table S2). The second stage involved comparing the chemical shift values for the unknown cross-peaks with the data obtained from the model spectra. The maximum permissible deviation was set at 1 and 5 ppm for ^1^H and ^13^C dimensions, respectively.

A quantitative analysis of the volume integrals of the HSQC correlation peaks was performed using Bruker’s Topspin 3.2 processing software. The relative abundances of side chains involved in the different inter-unit linkages were estimated in the aliphatic oxygenated region from the Cα–Hα correlations, except for β−O−4 substructures (A) for which Cβ–Hβ correlations were used. In the aromatic/unsaturated region, C_2_–H_2_ correlations from H, G and S lignin units were used to estimate their relative abundances [33].

### 2.5. Analytical Pyrolysis

Analytical pyrolysis was performed as described previously [15] using a GCMS-QP2010 Plus gas chromatography–mass spectrometry system (Shimadzu, Kyoto, Japan) equipped with an EGA/PY-3030D pyrolyzer (Frontier Lab, Koriyama, Japan) with a liquid nitrogen cooled cryo-trap. Lignin sample (~100 μg) was subjected to the thermal decomposition at 450 °C for 30 s in inert (6.0 grade helium) atmosphere with cryo-focusing of the formed products. Their chromatographic separation was achieved on an HP-5MS fused silica capillary column, 30 m × 0.25 mm i.d., with stationary phase film thickness 0.25 µm (Agilent, Santa Clara, CA, USA). High-purity helium (6.0 grade) was used as a carrier gas with a flow rate of 1 mL min^−1^. The temperature of the injector was 270 °C. The column temperature was programmed from 40 to 340 °C with a linear ramp of 10 °C min^−1^. The mass spectrometric detection was carried out with electron ionization (70 eV) in the scan mode (*m*/*z* 15–400). The ratio of S, G, and H structural units (S/G/H) was determined as the ratio of total areas of chromatographic peaks of monomeric compounds belonging to the corresponding classes. Their identification was performed using the Wiley and the NIST-2014 databases.

### 2.6. Atmospheric Pressure Photoionization High-Resolution Orbitrap Mass Spectrometry

Mass spectra were recorded on a Q Exactive Plus mass spectrometer (Thermo Scientific, Waltham, MA, USA) with a quadrupole mass filter and an orbital ion trap mass analyzer at a resolving power 70,000 (FWHM, at *m*/*z* 200) ensuring the screening studies and reliable determination of the elemental composition of lignin oligomers with molecular weights up to 600 Da [34]. An Ion Max ion source in APPI configuration equipped with a krypton gas discharge lamp with a quantum energy of 10.0 (10.6) eV was used. The mixture of 1,4-dioxane and water (90:10) was used as a mobile phase at a flow rate of 200 μL min^−1^ generated by an LC-30 HPLC system (Shimadzu, Kyoto, Japan). Lignin sample solution (5 μL) with a concentration of 50 mg L^−1^ was introduced into the ion source with a mobile phase by direct flow injection method without chromatographic separation [20]. The optimum parameters of the ion source ensured that the maximum intensity of mass spectra were applied: drying gas pressure 25 psi; flows of nebulizing gas and curtain gas 10 and 2 arbitrary units, respectively; temperature of desolvation line 250 °C; injector temperature 500 °C; radio-frequency voltage on the S-lens 55 arbitrary units. Mass spectra were recorded in the *m*/*z* range of 300–1500 with subsequent averaging of the results of at least ten measurements and subtracting the mobile phase background signal. The peak picking procedure was performed using relative intensity threshold value of 0.1% and signal-to-noise ratio of at least 10. Mass scale calibration was carried out prior to each analysis using a Pierce standard mixture (Thermo Scientific, Waltham, MA, USA) to ensure long-term mass accuracy < 3 ppm. Tandem mass spectra were recorded using collision-induced dissociation (CID) with nitrogen as a collision gas. For CID, 10–30 eV collision energies were applied.

Mass spectrometer control, data collection and primary processing were performed using Xcalibur software (Thermo Scientific, Waltham, MA, USA). The elemental compositions of lignin oligomers were determined based on accurate masses applying the following constrains: minimum degree of unsaturation (ring and double bond equivalent, RDB)—8; maximum number of C, H, and O atoms—100, 200 and 50, respectively; *m*/*z* range—300–1000. Home-made Microsoft Excel based software was used to filter data and build van Krevelen diagrams.

## 3. Results and Discussion

### 3.1. Dioxane Lignin Monomeric Units Identified by Py-GC/MS

Analytical pyrolysis allows one to obtain general information on the most abundant lignin macromolecule structural units based on the data of their thermal degradation products. A total of eighty-one compounds listed in the Supplementary Material (Table S3) were found in the Py-GC/MS chromatograms of the studied lignin samples. Among them, along with S, G, and H type structures typical for lignin, seven catechol derivatives (C-type structures) sourced primarily from tannins were identified. The contents of structures of each type were estimated using the chromatographic peak areas of the corresponding pyrolysis products, including catechin structures (Table 3). It can be seen that phloem lignin, regardless of whether it has undergone alkaline hydrolysis, demonstrates similar ratios of S and G type units (S/G) predominating in its structure. However, it sharply differs in this indicator from the xylem lignin in which the S/G ratio is more than twice as high. The fraction of C-units turned out to be significant, twice as high as the H-units content. Pyrolysis products of DL-P-AH and DL-X preparations also contained catechols (3.9 and 2.9%, respectively), although their proportion was expectedly much lower, and carrying out alkaline hydrolysis made it possible to reduce the content of C-type structures in phloem lignins by 40%. The observation of residual detectable amounts of C-type structures can be explained by the occurrence of side reactions of methoxy groups demethylation during pyrolysis as noted in the literature [35]. The achieved high degree of tannin removal during alkaline hydrolysis is also evidenced by the color change of the phloem lignin preparation from pinkish to light brown, characteristic of typical lignins (Supplementary material, Figure S2).

### 3.2. Chemical Composition and Structure of Lignin Analyzed by 2D-NMR

**Figure 1:**
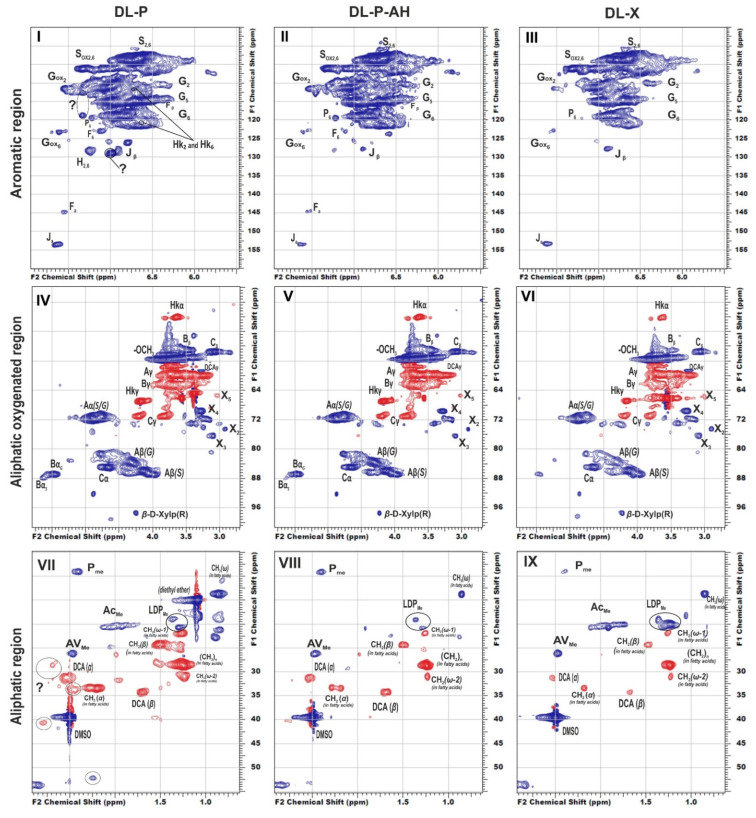
Key regions of HSQC spectra of birch lignin: DL-P (**I**,**IV**,**VII**); DL-P-AH (**II**,**V**,**VIII**); DL-X (**III**,**VI**,**IX**). The signal assignments are listed in Table S1. The main lignin structures identified are depicted in Figure 2.

**Figure 2:**
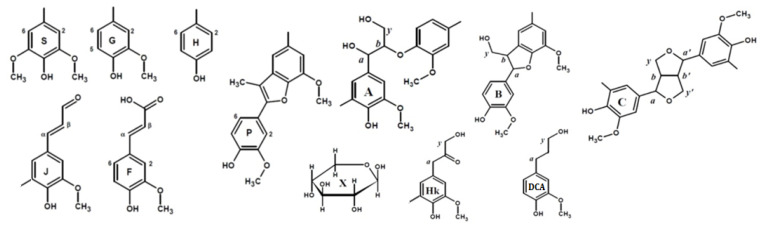
Principal structures and components present in the dioxane lignins of birch phloem as identified in the 2D HSQC NMR spectra A: β–aryl ethers; B: phenylcoumaran; C: resinols; Hk: Hibbert’s ketone; P: methyl-substituted phenylcoumarone; DCA: dihydroconiferyl alcohol; J: cinnamaldehyde end-groups; F: ferulic acid; X-β-D-Xylopyranose; H: hydroxyphenyl units; G: guaiacyl units; S: syringyl units.

To reveal structural features of birch phloem lignin, the obtained DL samples were analyzed by 2D NMR, which provides information on the main structural units of biopolymer macromolecule as well as the linkages between them. The main regions of the HSQC NMR spectra of the studied DL preparations, assigned cross-peaks and related structures are presented in Figure 1, Table S1 (Supplementary material), and Figure 2, respectively.

In general, the profiles of the ^1^H-^13^C HSQC NMR spectra of phloem dioxane lignin obtained both without and after alkaline hydrolysis do not differ substantially, and the nature of the detected cross-peaks fully corresponds to the spectral pattern of DL-X preparation (Figure 1). In particular, the main clusters of cross-peaks are observed in the aromatic region of the spectra and resonate from the atoms of the S- and G-type phenylpropane units (Figure 1, I–III). For both, the weak field region contains additional cross-peaks belonging to their oxidized fragments (indicated as S_ox_ and G_ox_). There is also a group of low-intensity cross-peaks arising from methyl-substituted phenylcoumarone (a degradation product of the phenylcoumaran fragment, designated as P) and cinnamic aldehyde (J). In the spectra of birch phloem lignins, cross-peaks of ferulic acid (F) were also detected.

In good agreement with Py-GC/MS data, S/G ratios calculated for phloem lignins were three-fold lower than for DL-X preparation, while absolute values of this parameter were noticeably overestimated (by 20% and 40% for phloem and xylem lignins, respectively) compared to those obtained by analytical pyrolysis (Table 4). The latter fact can be explained by a rather limited number of target analytes used for calculations in Py-GC/MS as well as by the possibility of partial demethoxylation of lignin under harsh conditions of pyrolysis. Nevertheless, the most important distinguishing feature of birch phloem lignin is the predominance of guaiacyl structures, which is uncharacteristic of xylem. This conclusion is confirmed by the published data [7] on the increase in the S/G ratio during the transition from cork (0.1) to phloem (0.7) and further to xylem (1.2) on the example of oak wood. Such a difference can be associated with different types of lignified cells in xylem and phloem tissues. The latter contains mainly sclereids and primary phloem fibers (Figure S3), which require more condensed lignin and thus more fractions of guaiacyl units capable of forming C-C bonds at the fifth position of the phenylpropane aromatic nucleus [7] to ensure optimal mechanical and protective properties [36].

**Figure 3:**
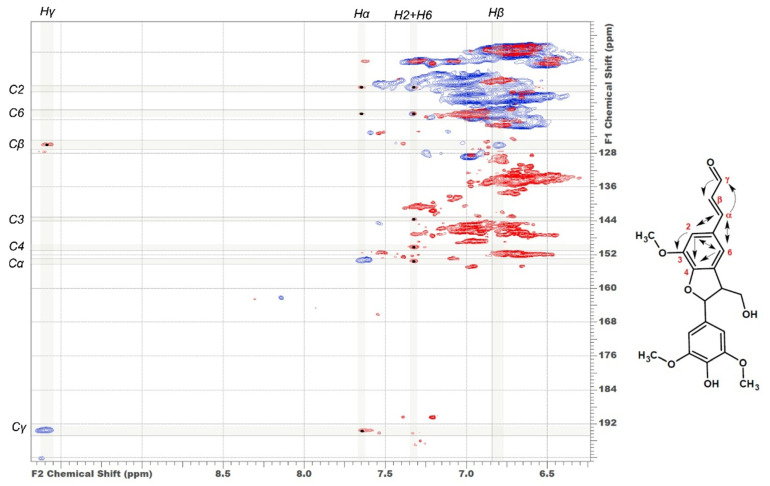
Comparison of HSQC (blue cross-peaks) and HMBC (red cross-peaks) spectra. Correlations used to identify the structure of 5-methoxybalanopholin are shown.

In addition to characteristic cross-peaks of the aromatic component, the spectrum of DL-P preparation obtained without preliminary alkaline hydrolysis possesses several distinctive features. First, these include cross-peaks at 112.5/7.30 and 118.8/7.30 ppm, which in a number of studies [4,7,29,30] were assigned to the structure of coniferyl aldehyde despite significant deviations in chemical shifts. The correlations present on the HMBC spectrum allowed for the obtainment of a more detailed characterization and assignment of a set of cross-peaks at 52.6/3.59, 87.7/5.62, 112.5/7.30, 118.8/7.30, 126.0/6.79, 153.4/7.64, and 193.8/9.61 ppm to the 5-methoxybalanopholin structure (Figure 3). This compound belonging to the above-mentioned phenylcoumarane structures is also known as biologically active neolignan [37]. The absence of its cross-peaks in the DL-P-AH spectrum suggests that it can be present in the DL-P preparation in the free form or as a part of the structure of condensed tannins co-extracted with lignin. A similar structure differing only in the absence of one methoxy group (guaiacyl moiety instead of syringyl one) was proposed earlier [19] as one of the most abundant dimers and base units for homologous series of oligomers in the composition of spruce dioxane lignin based on high-resolution mass spectrometry data.

**Figure 4:**
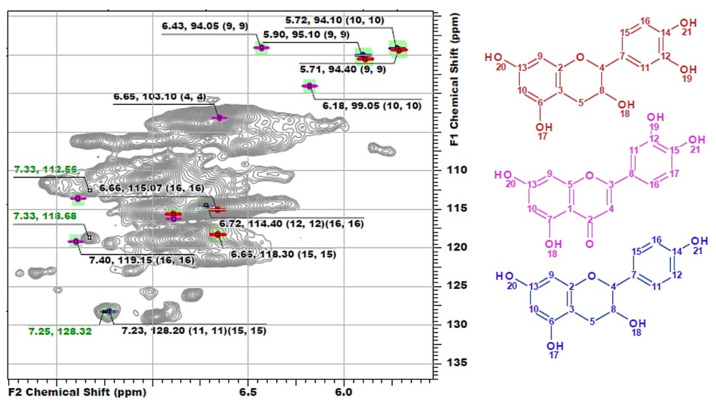
The comparison of model ^1^H-^13^C HSQC spectra of catechin (red), luteolin (pink) and afzelechin (blue) with the obtained spectrum of DL-P preparation.

Another distinguishing feature is the cross-peaks in the range of 127–130/6.8–7.4 ppm typical for both H-units and signals of double bonds in the structure of stilbenes. However, the measured exact values of the chemical shifts had significant deviations from the literature data on the representatives of these classes (Table S1). Since DL-P-AH and DL-X 2D NMR spectra do not contain such signals, it can be assumed that they belong to tannins or HPA extracted along with lignin or residues of low-molecular weight extractives, which were not removed during the sample preparation procedure. Long- and short-range correlation spectra did not allow for reliable identification of these compounds due to complexity of lignin spectra and low intensity of the related cross-peaks. In this regard, the method of constructing calculated spectra was used. Several representatives of each class of flavonoids (Figure S1) were chosen as target compounds for which model spectra were built. For flavonoids of each type, the standard deviations of the calculated chemical shifts in ^1^H and ^13^C dimensions were in the ranges of 0.00–0.53 and 0.07–0.97 ppm, respectively (Table S2). Further identification was carried out by visual comparison of the calculated spectra with the experimental one obtained for the DL-P sample (Figure 4).

The best match is achieved for the structure of luteolin, while cross-peaks observed at 112.53/7.33 and 118.69/7.33 ppm can also resonate from the catechin ring structure of phlobaphenes, and the cross-peak at 128.42/7.25 ppm can be attributed to the atoms of H-units in the structure of afzelechin. However, resonances from atoms in the composition of phenylalanine are also characterized by similar chemical shifts and were found repeatedly in the HSQC spectra of lignin, including in the preparation isolated from willow phloem [8]. In addition, the presence of catechin structures with cross-peaks overlapping with the signals of lignin G-units also cannot be ruled out. Evtuguin et al. [5,16,38] also repeatedly reported the presence of procyanidins in lignin preparations and attributed to them cross-peaks in the range of 110–125/6.5–6.8 ppm. However, in other sources, it was precisely established that, in this range of chemical shifts, G-ring atoms resonate as part of the structure of Hibbert ketones (Hk) [31]. According to the model spectra calculated in this work, the cross-peaks of catechin structures resonate in the range of 114–118/6.65–6.72 ppm. The absence of cross-peaks from atoms of phloroglucinol units in the spectrum can be explained by the fact that they participate in the formation of C–C bonds forming a high molecular weight polymer, which is consistent with the common concept of the structure of polyphenolic acids [13]. Thus, NMR spectroscopy, despite the detection of signals characteristic of flavonoids, did not allow for the obtainment of an unambiguous answer to the question about their presence in phloem lignin and about identifying specific compounds. The solution to this problem requires the use of mass spectrometric methods, which ensure the obtainment of more detailed information at the molecular level (see Section 3.3).

Cross-peaks in the aliphatic oxygenated region (Figure 1, IV–VI) and their integral values also indicate that the studied lignins do not have obvious qualitative differences in the fragment composition. All spectra show a number of characteristic cross-peaks belonging to fragments of β-aryl ether (A) and their degradation products (Hibbert ketones, labeled as Hk), resinol (C), and phenylcoumaran (B). A set of cross-peaks characteristic of β-D-xylopyranose (X) is also observed. An increased proportion of guaiacyl units and, as a result, a higher degree of condensation of phloem lignin is also expressed in the ratio of inter-unit linkages (Table 4). Thus, DL-P and DL-P-AH preparations expectedly contain less β-O-4′ alkyl-aryl ether bonds and three times more phenylcoumarane-type condensed structures. A number of distinctive cross-peaks in the range of 25–45/2.5–3.0 ppm can also be seen in the HSQC spectra. Analysis of the literature data showed that they can be attributed to the variety of phenolic compounds with saturated propane chains, including platyphylloside-like structures [39] with carbohydrate moiety, typically present in the phloem along with tannins and flavonoids. Analysis of unidentified cross-peaks using an ACD/Labs expert system confirmed this conclusion and revealed a good match in chemical shifts with the cross-peaks in the aromatic region (Figure S4).

In the aliphatic regions of the spectra (Figure 1, VII–IX), cross-peaks resonating from fatty acids, dihydroconiferyl alcohol (DCA) as well as methyl groups in acetovanillone (AV) structure and other lignin degradation products (LDP) are observed. The presence of cross-peaks from DCA is unusual for hardwood lignins, since these terminal units are typical for softwood lignins [40]. However, the presence of these fragments in the birch xylem lignin was also observed earlier in [41].

### 3.3. Molecular Level Characterization by High-Resolution Mass Spectrometry

Atmospheric pressure photoionization mass spectra of the three studied lignin preparations (Figure S5) contain up to 3000 peaks corresponding mainly to deprotonated molecules ([M − H]^−^) in the *m*/*z* range of 300–1500 and are characterized by a similar general appearance. Four broad peak clusters corresponding to lignin oligomers with different degrees of polymerization (from dimers at *m*/*z* 300–450 to pentamers at *m*/*z* 950–1200) can be visually distinguished. Their centers are separated from each other by a distance of 200–250 Da typical for lignins of deciduous wood with a high content of S-units. A distinctive feature of the phloem lignin is the presence of high-molecular fraction with signals in an *m*/*z* range of 700–1300. This is consistent with the shown-above predominantly guaiacylic nature of phloem lignin and, therefore, its higher degree of condensation. Another reason closely related to the higher capability of condensation is an increased content of C-C inter-unit linkages and phenylcoumarane structures that are much more stable under conditions of APPI compared to easily destructible alkyl-aryl ether bonds [20]. Two basic representatives of phenylcoumarane compounds—balanopholin and identified in NMR experiments methoxybalanopholin (Figure 3)—give intense signals in the mass spectra of phloem lignin samples at *m*/*z* 355.1199 ([C_20_H_19_O_6_]^−^) and 385.1294 ([C_21_H_21_O_7_]^−^), which are more than one and a half times higher in relative intensity than the same peaks in the DL-X spectrum (Table S4).

**Figure 5:**
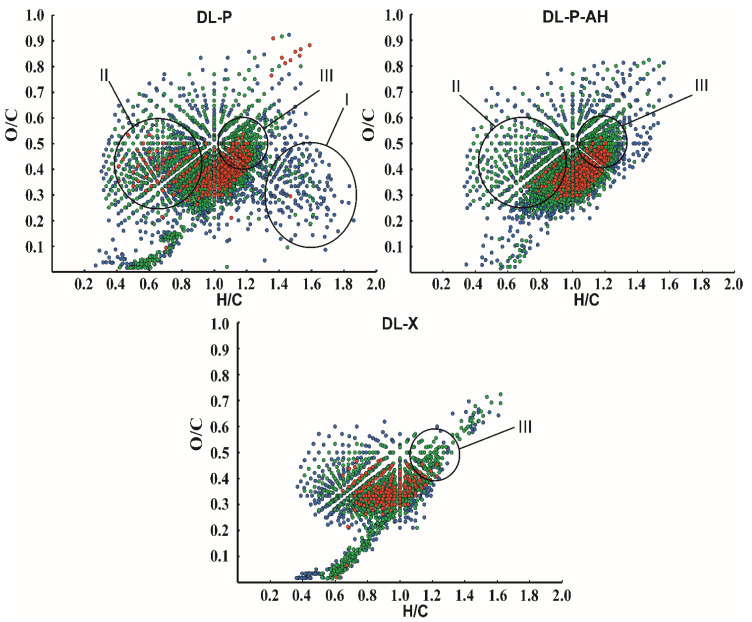
Van Krevelen plots for DL-P, DL-P-AH and DL-X lignin preparations obtained from negative ion mode APPI-Orbitrap mass spectra. The color coding indicates the relative intensity of the signals in the mass spectrum: red, 100–10%; green, 10–1%; and blue, 1–0.1%.

A more detailed picture of lignin chemical composition as a complex object at the molecular level was obtained by visualizing the elemental ratios of all detected compounds in van Krevelen coordinates (Figure 5). The obvious difference in the DL-P sample from others is the presence of a large number of signals from structures with a low degree of unsaturation (region I, H/C = 1.4–1.8), as well as a set of intense peaks in region II. The first can be easily attributed to lipid and fatty acid impurities, while the latter correspond mainly to polyphenolic substances. Among them, the signals at *m*/*z* 301.0354 and 303.0511 with corresponding elemental compositions [C_15_H_9_O_7_]^−^ and [C_15_H_11_O_7_]^−^, respectively, as well as some of their methylated derivatives (additional methylene group) predominate. They were attributed to quercetin and dihydroquercetin, and the reliability of quercetin identification was confirmed by tandem mass spectrometry data and their comparison with the spectrum of analytical standard (Table S5). Ions corresponding to these structures were also observed in DL-P-AH and DL-X mass spectra; however, their intensities were 4–5-fold lower than in DL-P spectrum. Although quercetin was not included in the list of the most probable flavonoid components in phloem lignin according to NMR data, its structure is quite close to those proposed in Figure 4. Both of these areas in the van Krevelen plot disappear after alkaline treatment of the phloem, making the “image” of the DL-P-AH chemical composition closer to that of the xylem lignin preparation. A distinctive feature of both phloem lignin preparations is the presence of region III (H/C = 1.0–1.3, O/C = 0.4–0.6), with intense signals from compounds with a higher oxygen content and somewhat lower unsaturation compared to typical lignin oligomers isolated from birch xylem. The search for the corresponding peaks in the mass spectra showed that they belong mainly to compounds with higher molecular weights (>600 Da). Based on elemental compositions, they can be attributed to quaiacylglycerol structures [19] or glycosylated lignin oligomers.

The possibilities of tandem mass spectrometry for the study of individual compounds in the composition of lignin are limited by the presence of many isomeric and isobaric structures falling within the isolation window of quadrupole mass filter. However, important structural information can be obtained using broadband CID, which implies the fragmentation of all precursor ions in a wide *m*/*z* range. Application of this technique for precursor ions with molecular weights of 300–1200 Da allowed for the detection of carbohydrates among the resulting monomeric fragments (Table S6). Pentoses with the gross formula C_5_H_10_O_5_ (*m*/*z* 149.0454 for [M − H]^−^) are distinguished by the highest signal intensity, which is consistent with the detection in the aliphatic oxygenated region of the NMR spectrum of β-D-xylopyranose. This indicates the possibility of an increased content of lignin–carbohydrate complexes in the phloem lignin. On the contrary, flavonoids and their typical fragments were not found among the broadband CID products (precursor ions *m*/*z* range 500–1200) of phloem lignin. This suggests that they are present in the studied lignin preparations in the form of low-molecular impurities that are not incorporated into the structure of lignin macromolecules. The composition of monomeric products of CID of lignin oligomers fully confirms the conclusion about the significant predominance of guaiacyl structures in phloem lignin compared to preparation isolated from xylem.

## 4. Conclusions

The combination of two-dimensional NMR spectroscopy and atmospheric pressure photoionization high-resolution mass spectrometry for the first time made it possible to obtain valuable information on the chemical composition and structure of the poorly studied lignin of birch phloem, which is a large-tonnage byproduct of wood processing and a promising feedstock for biorefining. Birch phloem and xylem lignins are characterized by a similar set of principal structural elements due to the identity of precursors (monolignols) involved in their formation, both in phloem sclereids and xylem fibers. The most important distinguishing feature of phloem lignin is the predominance of guaiacyl structures over syringyl ones (S/G = 0.7–0.9) providing a higher degree of condensation due to the formation of C-C bonds involving the 5th position in the phenylpropane aromatic nucleus. The main types of inter-unit linkages are β-O-4′ alkyl-aryl ether followed by phenylcoumaran and resinol structures, while in some cases, cinnamic aldehyde and dihydroconiferyl alcohol act as terminal units. The complexity of the chemical composition of dioxane lignin preparations isolated from the phloem is also associated with the presence of flavonoids (condensed tannins) and lipids (fatty acids), which can be removed during the preliminary alkaline hydrolysis of plant material. The use of high-resolution tandem mass spectrometry allowed for the demonstration of the fact that flavonoids are not incorporated into the lignin structure and can be considered as impurity compounds extracted together with lignin from phloem tissues. A significant role in the formation of the structure of birch phloem lignin is played by lignin–carbohydrate complexes, in which β-D-xylopyranose moieties predominate.

## Figures and Tables

**Table 1:** Chemical composition of the birch phloem under study.

Parameter	Content, % (Recalculated to Dry Material)	
	Experimental Data	Literature Data [25]
Total extractives	16.7	14.6
Klason lignin after Soxhlet extraction	28.5	-
Substances extracted with 1% NaOH	21.2	23.8
Klason lignin after Soxhlet and 1% NaOH extractions	22.5	19.5

**Table 2:** Molecular weight characteristics and functional composition of the obtained lignin preparations.

Parameter	Lignin Sample		
	DL-P	DL-P-AH	DL-X
M_w_, Da	4350	4400	4400
M_n_, Da	2200	1500	1700
PDI	1.97	2.93	2.59
OH_phen_, %	4.4	3.8	2.8
OH_aliph_, %	7.9	7.8	7.4
COOH, %	0.33	0.33	0.18

**Table 3:** Relative molar abundance (%) of the structural units of different types and S/G ratio for the birch phloem and xylem dioxane lignin preparations determined by Py-GC/MS.

Structure Type	DL-P	DL-P-AH	DL-X
S	38	40	63
G	52	54	33
H	3	2	1
C	6	4	3
S/G	0.73	0.74	1.91

**Table 4:** Abundance of inter-unit linkages and S-, G-, H-type structures in lignin preparations (per 100 phenylpropane units) according to NMR spectroscopy analysis.

Structure	DL-P	DL-P-AH	DL-X
β-O-4′ (β-aryl ether), including:	35.9	34.3	42.5
between G- and S-type units, A_S_	20.2	19.1	27.8
between G-type units; A_G_)	10.5	10.1	8.0
α-O-4 (Phenylcoumarane)	4.7	4.3	1.4
α-O-γ’ (Resinol)	8.1	7.4	7.3
S	43.5	46.1	73.3
G	54.5	53.9	26.7
H	2.0	0.0	0.0
S/G	0.87	0.86	2.74

## Data Availability

The data presented in this study are available in the article and Supplementary Materials.

## Supplementary Materials

The following are available online at https://www.mdpi.com/article/10.3390/polym14050964/s1. Figure S1: Model (calculated) spectra of various representatives of the flavonoid class; Figure S2: Appearance of the studied samples; Figure S3: Lignified anatomical elements of birch phloem; Figure S4: Comparison of the calculated ^1^H-^13^C HSQC spectrum of platyphyloside with the experimental spectrum for birch phloem; Figure S5: APPI-Orbitrap-HRMS mass spectra of DL-P. DL-P-AH and DL-X; Table S1: Assignments of the ^1^H-^13^C cross-peaks in the HSQC spectra (in DMSO-d6) of the dioxane lignin from the birch phloem and xylem; Table S2: Error of chemical shifts for model spectra of various representatives of the flavonoid class; Table S3: Identities of the Lignin-Derived Compounds Identified in Py-GC/MS of the Birch Lignin Dioxane; Table S4: Relative intensities of balanofolin and methoxybalanofolin signals in birch xylem and phloem mass spectra; Table S5: Tandem mass spectra of precursor ion with *m*/*z* 301 in DL-P mass spectrum and quercetin analytical standard (collision energy 20 eV); Table S6: Major monomeric compounds tentatively identified in broadband CID tandem mass spectra of birch xylem and phloem lignin preparations (precursor ion *m*/*z* range 300–1000, collision energy 10 eV).
