# Supplementary material for: Features of the Chemical Composition and Structure of Birch Phloem Dioxane Lignin: A Comprehensive Study
Source: Polymers (Basel). 2022 Feb 28;14(5):964. doi: 10.3390/polym14050964 (PMC8912895; doi:10.3390/polym14050964)
Supplement: Supplementary file 1 [file polymers-14-00964-s001.zip › polymers-1615931-supplementary.pdf]

Supplementary
Features of the Chemical Composition and Structure of Birch Phloem Dioxane Lignin: A Comprehensive Study

Anna V. Faleva*, Ilya I. Pikovskoi, Sergey A. Pokryshkin, Dmitry G. Chukhchin and Dmitry S. Kosyakov*

Laboratory of Natural Compounds Chemistry and Bioanalytics, Core Facility Center "Arktika", M.V. Lomonosov Northern (Arctic) Federal University, Northern Dvina Emb. 17, Arkhangelsk, 163002, Russia; a.bezumova@narfu.ru (A.V.F.), i.pikovskoj@narfu.ru (I.I.P.), s.pokryshkin@narfu.ru (S.A.P.), d.chuhchin@narfu.ru (D.G.Ch), d.kosyakov@narfu.ru (D.S.K.)

*Correspondence: a.bezumova@narfu.ru (A.V.F.), d.kosyakov@narfu.ru (D.S.K.)

Abstract: Understanding the chemical structure of lignin in the plant phloem contributes to the systematics of lignins of various biological origins, as well as the development of plant biomass valorization. In this study, the structure of the lignin from birch phloem has been characterized using the combination of three analytical techniques, including 2D NMR, Py-GC/MS, and APPI-Orbitrap-HRMS. Due to the specifics of the phloem chemical composition, two lignin preparations were analyzed: a sample obtained as dioxane lignin (DL) by the Pepper's method and DL obtained after preliminary alkaline hydrolysis of the phloem. The obtained results demonstrated that birch phloem lignin possesses a guaiacyl–syringyl (G-S) nature with a unit ratio of (S/G) 0.7–0.9 and a higher degree of condensation compared to xylem lignin. It was indicated that its macromolecules are constructed from β-aryl ethers followed by phenylcoumaran and resinol structures as well as terminal groups in the form of cinnamic aldehyde and dihydroconiferyl alcohol. The presence of fatty acids and flavonoids removed during alkaline treatment was established. Tandem mass spectrometry made it possible to demonstrate that the polyphenolic components are impurities and are not incorporated into the structure of lignin macromolecules. An important component of phloem lignin is lignin–carbohydrate complexes incorporating xylopyranose moieties.

Keywords: birch phloem, lignin, nuclear magnetic resonance, high-resolution mass spectrometry, Py-GC/MS

Table S1. Assignments of the ^1H-^{13}C cross-peaks in the HSQC spectra of the dioxane lignin from the birch phloem and xylem.

Labels	Substructures		δC/δH correlations (ppm)														
			Experimental data												Literature data [18, 28, 31]	
			DL-P				DL-P-AH				DL-X					
			δC	Δ	δH	Δ	δC	Δ	δH	Δ	δC	Δ	δH	Δ	δC	δH
A	β-aryl ether	α (G)	71.20	0.30	4.76	0.05	71.11	0.21	4.75	0.04	71.19	0.29	4.75	0.04	70.9	4.71
		α (S)	71.68	0.12	4.88	0.05	71.64	0.16	4.88	0.05	71.75	0.05	4.87	0.04	71.8	4.83
		β (G)	83.53	0.37	4.31	0.03	83.50	0.40	4.31	0.03	83.49	0.41	4.31	0.03	83.9	4.28
		β (G/S)	85.85	0.05	4.14	0.02	85.85	0.05	4.13	0.01	85.81	0.09	4.13	0.01	85.9	4.12
		β (S)	86.77	0.34	4.02	-	86.65	0.46	4.01	-	86.52	0.59	4.02	-	87.11	-
		γ	59.78	0.02	3.63	0.02	59.75	0.05	3.63	0.02	59.77	0.03	3.64	0.03	59.8	3.61
		γ2			3.25	0.01			3.24	0.00			3.22	0.00		3.24
B	phenylcoumaran	α	86.77	0.03	5.47	0.04	86.78	0.02	5.47	0.04	86.76	0.04	5.47	0.04	86.8	5.43
		β	53.11	0.01	3.48	0.05	53.08	0.02	3.48	0.05	53.18	0.08	3.47	0.04	53.1	3.43
		γ	62.38	0.22	3.74	0.01	62.43	0.17	3.72	0.01	62.29	0.31	3.75	0.02	62.6	3.73
		γ2			-	-			-	-			-	-		3.62
C	Resinol	α	84.88	0.08	4.68	0.03	84.93	0.13	4.67	0.02	84.82	0.02	4.66	0.01	84.8	4.65
		β	53.46	0.04	3.08	0.03	53.47	0.03	3.07	0.02	53.46	0.04	3.06	0.01	53.5	3.05
		γ	70.95	0.05	4.2	0.03	71.03	0.13	4.19	0.02	70.93	0.03	4.19	0.02	70.9	4.17
		γ2			3.81	0			3.81	0			3.81	0		3.81
J	Cinnamyl aldehyde	α	153.47	0.07	7.64	0.03	153.53	0.13	7.62	0.01	153.26	0.14	7.63	0.02	153.4	7.61
		β	126.07	0.23	6.79	0.03	**127.76**	**1.46**	**6.90**	**0.14**	**127.74**	**1.44**	**6.90**	**0.14**	126.3	6.76
		γ	193.87	0.07	9.61	0.06	194.02	0.22	9.62	0.05	193.97	0.17	9.62	0.05	193.8	9.67
		6	**118.80**	**5.06**	**7.30**	**0.09**	-	-	-	-	-	-	-	-	123.86	7.21
Hk	Hibbert's ketone	α	44.14	0.16	3.65	0.03	44.09	0.21	3.64	0.02	44.18	0.12	3.64	0.02	44.3	3.62
		γ	66.98	0.32	4.17	0.02	66.93	0.37	4.17	0.02	66.91	0.39	4.16	0.01	67.3	4.15
DCA	dihydroconiferyl alcohol	α	31.18	0.16	**2.54**	**0.10**	31.15	0.19	2.53	0.11	31.24	0.1	**2.53**	**0.11**	31.34	2.64*
		β	34.30	0.29	**1.70**	**0.11**	34.25	0.34	1.69	0.12	34.34	0.25	**1.68**	**0.13**	34.59	1.81*
P	phenylcoumarone	γ	9.00	0.30	2.41	0.04	9.09	0.21	2.4	0.03	8.89	0.41	2.39	0.02	9.3	2.37
		G6	119.36	0.04	7.22	0.02	119.41	0.01	7.21	0.01	119.58	0.18	7.2	0	119.4	7.2
F	ferulic acid	α	144.77	0.07	7.55	0.03	144.74	0.04	7.55	0.03	-	-	-	-	144.70	7.58
		β	114.32	0.48	6.37	0.02	114.38	0.42	6.37	0.02	-	-		-	114.8	6.39
		6	123.00	0.28	7.11	0.05	123.1	0.38	7.11	0.05	-	-		-	122.72	7.16
	Stilbene	CH=CH	**128.90**	**2.75**	6.97	0.03	-	-	-	-	-	-	-	-	126.15	6.94
AV	acetovanillone	Me	26.22	0.00	2.47	0.1	26.25	0.03	2.47	0.1	26.25	0.03	2.47	0.1	26.22	2.57**

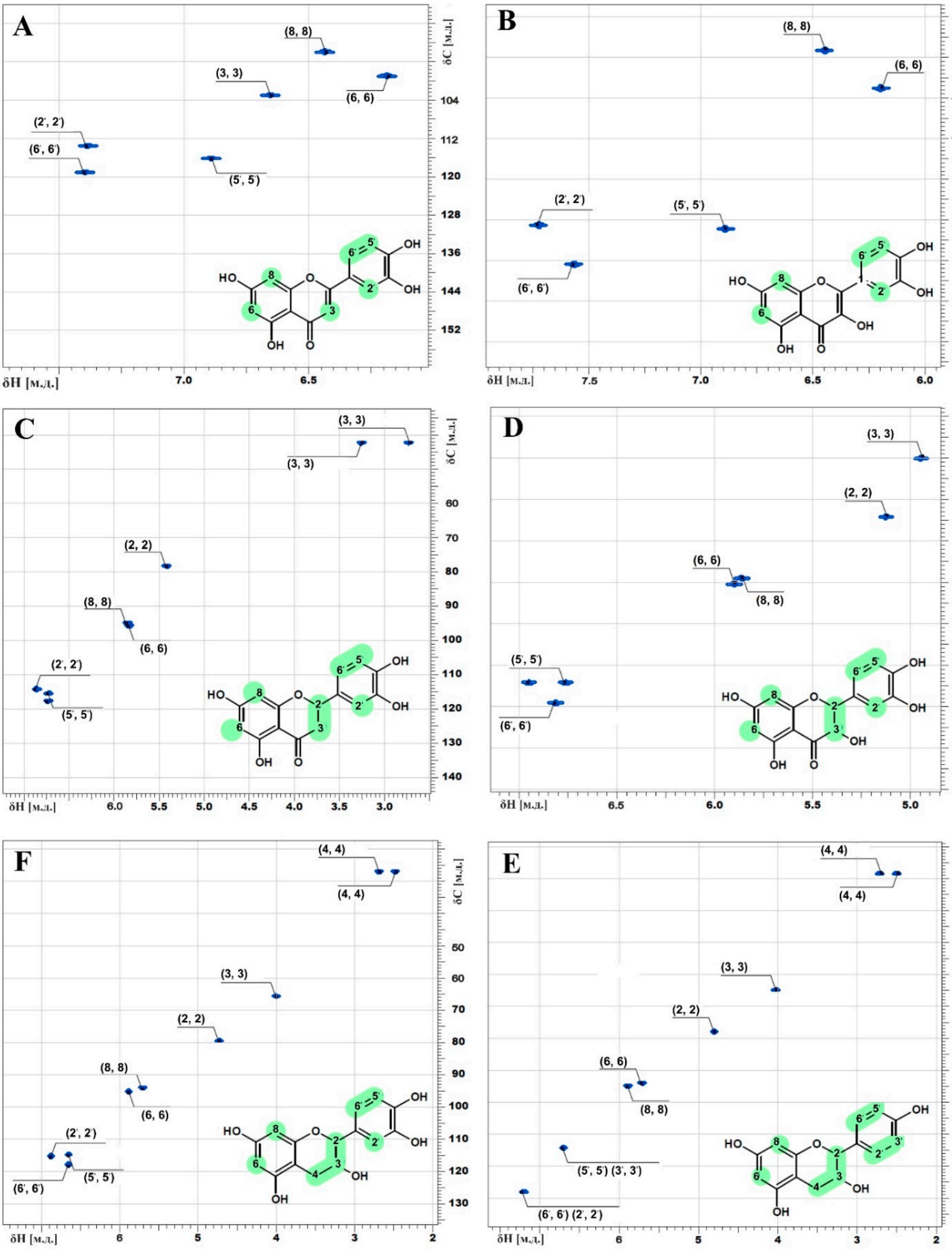

Figure S1. Model (calculated) spectra of various representatives of the flavonoid class.

Table S2. Error of chemical shifts for model spectra of various representatives of the flavonoid class.

Spectrum Label	Name of the structure	№	13C, ppm	Δ	1H, ppm	Δ
		Flavone				
A	Luteolin	C2’	113.60	0.26	7.39	0.00
		C5’	116.25	0.21	6.89	0.00
		C6’	119.15	0.21	7.40	0.00
		C3	103.10	0.28	6.65	0.00
		C8	94.05	0.21	6.43	0.00
		C6	99.05	0.21	6.18	0.00
		Flavonol				
B	Quercetin	C2’	115.55	0.95	7.73	0.38
		C5’	116.05	0.85	6.89	
		C6’	120.5	1.50	7.57	
		C6	98.65	0.95	6.19	
		C8	93.93	1.07	6.44	
		Flavonone				
C	Eriodictyol	C6’	117.85	0.07	6.75	0.38
		C5’	114.35	0.21	6.88	0.38
		C2’	115.45	0.21	6.74	0.38
		C2	78.35	0.07	5.42	0.48
		C3	42.20	0.00	2.72/3.25	0.53<br>0.53
		C6	95.80	0.14	5.85	0.38
		C8	94.95	0.21	5.87	0.38
		Flavanonols				
D	Dihydroquercetin	C6’	119.35	0.21	6.82	0.38
		C5’	115.35	0.07	6.95	0.38
		C2’	115.35	0.07	6.77	0.38
		C2	83.10	0.00	5.12	0.37
		C3	71.65	0.07	4.94	0.36
		C6	96.10	0.00	5.90	0.38
		C8	95.05	0.07	5.86	0.38
		Flavan-3-ol				
E	Catechin	C6’	118.30	0.50	6.66	0.00
		C5’	115.65	2.45	6.89	0.00
		C2’	115.07	0.33	6.66	0.00
		C2	79.65	2.19	4.73	0.00
		C4	27.07	1.97	2.47/2.68	0.00
		C3	65.85	1.06	4.00	0.00
		C6	95.50	0.10	5.89	0.00
		C8	94.40	0.10	5.71	0.00
F	Afzelechin	C3’	128.20	0.00	7.23	0.00
		C5’	128.20	0.00	7.23	0.00
		C2’	114.40	0.00	6.72	0.00
		C6’	114.40	0.00	6.72	0.00
		C2	78.00	0.00	4.80	0.00
		C4	28.20	0.00	2.48/2.69	0.00
		C3	64.80	0.00	4.02	0.00
		C6	94.10	0.00	5.72	0.00
		C8	95.10	0.00	5.90	0.00

Table S3. Identities of the Lignin-Derived Compounds Identified in Py-GC/ MS of the Birch Lignin Dioxane

peak	Rt	Name of compounds	Origin	DL-P	DL-P- AH	DL-X
1	13.07	Phenol	H	0.917	0.710	0.685
2	16.55	Phenol. 2-methyl-	H	0.246	0.222	0.191
3	17.57	Phenol. 4-methyl-	H	0.886	0.408	0.366
4	18.18	Phenol. 2-methoxy-	G	9.759	12.994	7.984
5	19.02	Phenol. 2.6-dimethyl-	H	0.039	0.042	0.024
6	20.44	Phenol. 2-methoxy-3-methyl	G	0.015	0.020	0.017
7	20.68	Phenol. 2.5-dimethyl-	H	0.010	0.010	0.003
8	21.18	Phenol. 2.4-dimethyl- + Phenol. 2.3-dimethyl-	H	0.331	0.191	0.104
9	22.09	Phenol. 3-ethyl-	H	0.147	0.055	0.031
10	22.22	Phenol. 4-ethyl-	H	0.063	0.066	0.043
11	22.62	Phenol. 2-methoxy-5-methyl	G	0.541	0.547	0.463
12	22.99	Phenol. 4-methoxy-3-methyl	G	0.074	0.125	0.112
13	23.33	Phenol. 2-methoxy-4-methyl	G	8.234	4.339	2.583
14	23.71	Catechol	C	3.447	1.807	0.981
15	25.09	Phenol. 3-methoxy-	G	0.127	0.112	0.084
16	25.57	Phenol. 4-ethyl-3-methyl-	H	0.043	0.023	0.020
17	25.92	Phenol. 3.6-dimethoxy-	S	0.007	0.017	0.035
18	26.58	Pyrogallol 1-methyl ether	C	1.922	1.771	1.799
19	26.68	Pyrocatechol. 3-methyl-	C	0.466	0.006	0.009
20	27.52	Phenol. 4-ethyl-2-methoxy- (Ethylguaiacol)	G	2.069	1.492	0.989
21	28.02	Resorcinol	C	0.006	0.029	0.006
22	28.07	Pyrocatechol. 4-methyl-	C	0.523	0.231	0.000
23	28.49	Phenol. 4-ethyl-2-methoxy-	G	0.003	0.014	0.008
24	29.16	2-Methoxy-4-vinylphenol	G	5.263	5.020	2.132
25	29.53	Phenol. 3-methoxy-5-methyl	G	0.375	0.231	0.206
26	29.65	Phenol. 3.4-dimethoxy-	S	0.000	0.017	0.018
27	30.44	Phenol. 3.4-dimethoxy-	S	0.104	0.062	0.000
28	30.95	Phenol. 2.6-dimethoxy- (Syringol)	S	6.709	9.986	13.413
29	31.15	Acetophenone. 2-hydroxy-5-methoxy-	G	0.025	0.019	0.044
30	31.19	Phenol. 2-methoxy-4-(2-propenyl)-	G	0.382	0.371	0.173
31	31.24	Phenol. 3.4-dimethoxy-	S	0.452	0.307	0.292
32	31.36	Acetophenone. 4-hydroxy-3-methoxy- (Acetovanillone)	G	0.102	0.063	0.032
33	31.63	Phenol. 2-methoxy-4-propyl- (Propylguaiacol)	G	0.422	0.372	0.230
34	31.84	Phenol. 4-propyl-	H	0.000	0.001	0.000
35	32.36	4-Ethylcatechol	C	0.000	0.000	0.004
36	32.91	Phenol. 2-methoxy-5-(1-propenyl)-	G	0.061	0.042	0.032
37	32.99	Benzaldehyde. 4-hydroxy-3-methoxy- (Vanillin)	G	1.326	1.221	1.528
38	33.18	Phenol. 2-methoxy-3-(1-propenyl)- (Isoeugenol)	G	0.038	0.024	0.014
39	33.45	Phenol. 2-methoxy-4-(1-propenyl)- (Isoeugenol)	G	0.254	0.268	0.129
40	33.57	Phenol. 5-methoxy-2.3-dimethyl-	G	0.089	0.043	0.031
41	35.22	Phenol. 4-methyl. 2.6-dimethoxy- (4-methylsyringol)	S	6.510	3.536	4.846
42	35.28	Phenol. 2-methoxy-4-propenyl- (cis-Isoeugenol)	G	2.290	2.294	1.073
43	35.70	Phenol. 2-methoxy-4-propyl-	G	3.362	3.280	2.533
44	36.82	Phenol. 2-methoxy-4-acetyl	G	1.313	1.174	0.586
45	37.05	Acetylguaiacol isomer	G	0.022	0.051	0.000
46	38.16	Benzoic acid. 4-hydroxy-3-methoxy-. methyl ester (Methyl vanillate)	G	0.153	0.266	0.122
47	38.58	4-ethyl-2.6-dimethoxy-phenol (4-ethyl-syringol)	S	1.053	0.748	1.346
48	38.75	4-(2-Hydroxyethyl)-2-methoxyphenol	G	3.355	3.516	2.431

49	39.82	Ethanone. 1-(4-hydroxy-3-methoxyphenyl)-	G	0.011	0.000	0.001
50	40.20	Phenol. 4-ethenyl. 2.6-dimethoxy (4-vinylsyringol)	S	4.169	4.088	5.946
51	40.54	4-((1E)-3-Hydroxy-1-propenyl)-2-methoxyphenol	G	0.944	0.783	0.474
52	40.79	Benzeneacetic acid. 4-hydroxy-3-methoxy-. methyl ester	G	0.058	0.145	0.127
53	40.89	propano 3-methoxy-4-hydroxyphenone	G	0.962	0.772	0.458
54	40.91	4-Hydroxy-2-methoxycinnamaldehyde	G	0.192	0.169	0.074
55	41.05	Ethanone. 1-(4-hydroxy-3-methoxyphenyl)- (Apocynin)	G	1.998	1.551	2.663
56	41.72	4-n-Propylresorcinol	C	0.059	0.063	0.090
57	41.71	Phenol. 4-(2-propenyl). 2.6-dimethoxy- (4-allylsyringol)	S	0.532	0.488	0.610
58	41.98	Phenol. 4-propyl. 2.6-dimethoxy-(4-propylsyringol)	S	0.229	0.179	0.281
59	42.68	Phenol. 4-(3-hydroxy-1-propenyl)-	G	0.006	0.000	0.000
60	43.41	(4-Hydroxy-3-methoxyphenyl)acetic acid (Homovanillic acid)	G	1.111	1.312	0.483
61	43.62	Phenol. 4-(2-propenyl). 2.6-dimethoxy- (4-propenylsyringol- cis)	S	0.359	0.354	0.417
62	43.93	Benzaldehyde. 4-hydroxy-3.5-dimethoxy- (Syringaldehyde)	S	1.689	1.809	4.182
63	44.30	4-((1E)-3-Hydroxy-1-propenyl)-2-methoxyphenol	G	0.378	0.634	0.207
64	44.96	1-Propanone. 3-hydroxy-1-(4-hydroxy-3-methoxyphenyl)-	G	0.159	0.049	0.655
65	45.06	3-Methoxy-4-hydroxybenzalacetone	G	0.054	0.038	0.017
66	45.53	Phenol. 2.6-dimethoxy-4-(2-propenyl)- (4-propenylsyringol- trans)	S	2.973	2.834	3.762
67	45.69	1-(4-Hydroxy-3.5-dimethoxyphenyl)-ethanal (Homosyringaldehyde)	S	2.013	2.121	5.165
68	46.39	Acetic acid. (4-hydroxy-3-methoxyphenyl)-. methyl ester (Methyl homovanillate)	G	0.889	0.532	0.989
69	46.66	2-Propenal. 3-(4-hydroxy-3-methoxyphenyl)- (Coniferyl aldehyde)	G	0.833	0.901	0.341
70	46.75	Acetophenone. 4-hydroxy-3.5-dimethoxy- (Acetosyringone)	S	1.925	1.486	1.905
71	46.90	2-Propen-1-ol. 3-(4-hydroxy-3-methoxyphenyl)- (Coniferyl alcohol)	G	5.133	9.583	2.669
72	47.76	2-Butanone. 4-(4-hydroxy-3-methoxyphenyl)- (Vanillylacetone)	G	0.108	0.081	0.118
73	48.17	Benzoic acid. 4-hydroxy-3.5-dimethoxy-. methyl ester (Syringic acid. methyl ester)	S	0.116	0.201	0.185
74	48.19	1-(4-Hydroxy-3.5-dimethoxyphenyl)-2-propanone (Syringyl acetone)	S	2.454	2.817	7.774
75	49.87	3-(4-Hydroxy-3.5-dimethoxyphenyl)-1-propanal	S	0.460	0.360	0.591
76	50.10	1-Propanone. 1-(4-hydroxy-3.5-dimethoxyphenyl) (Propiosyrongone)	S	1.723	1.130	4.816
77	50.25	1-Propen-2-al. 3-(4-hydroxy-3.5-dimethoxyphenyl) (Propenylsyringone)	S	0.139	0.125	0.190
78	52.40	1-(4-Hydroxy-3.5-dimethoxyphenyl)-1-propanol (Dihydrosinapyl alcohol)	S	0.349	0.403	0.311
79	53.19	2-Propen-1-ol. 3-(4-hydroxy-3.5-dimethoxyphenyl) (Sinapyl alcohol. cis)	S	0.299	0.490	0.380
80	55.51	Sinapic aldehyde	S	1.431	1.277	2.080
81	55.89	2-Propen-1-ol. 3-(4-hydroxy-3.5-dimethoxyphenyl) (Sinapyl alcohol. trans)	S	2.709	5.086	4.282

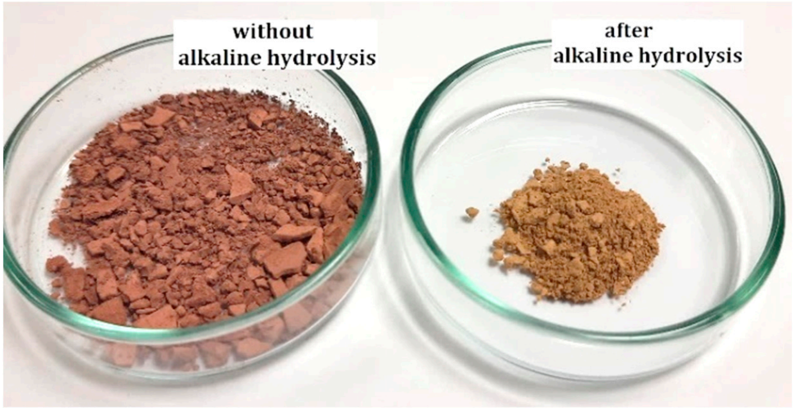

Figure S2. Appearance of the studied samples

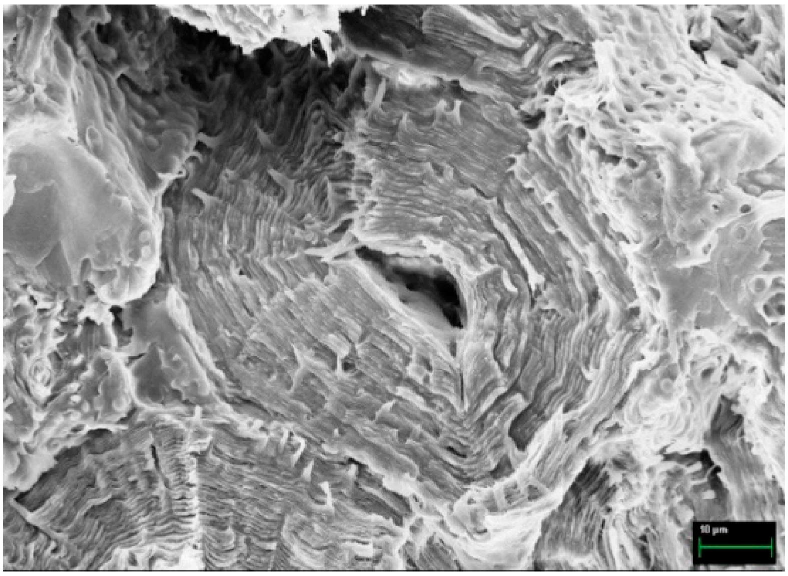

(a)

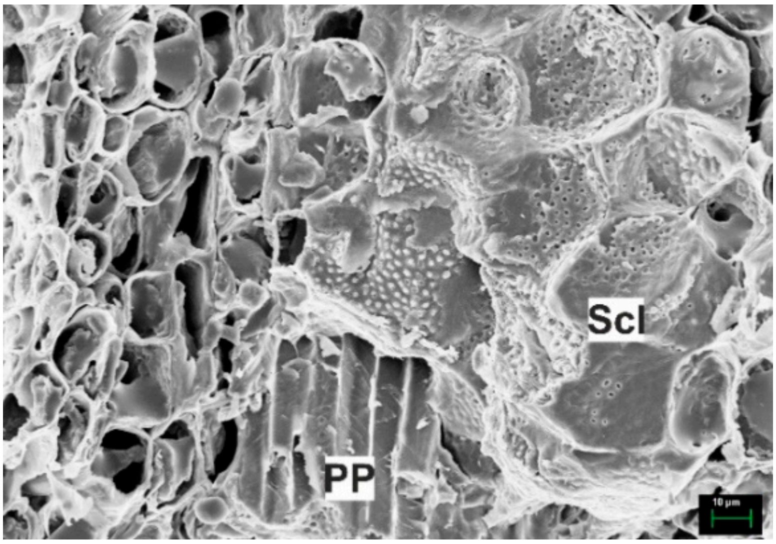

(b)

Figure S3. Lignified anatomical elements of birch phloem. Scale: 10 microns. a) Sclereids, b) sclereids (Scl) and primary phloem fibers (PP) (radial incision).

Atom#	C Shift	H Shift	C Calc Shift (HOSE)	H Calc Shift (HOSE)
4	28.621	2.690	29.130	2.834
10	40.660	2.790	44.090	2.684
27, 28	114.604	6.712	115.440	6.675
25, 26	128.923	6.976	129.440	7.047

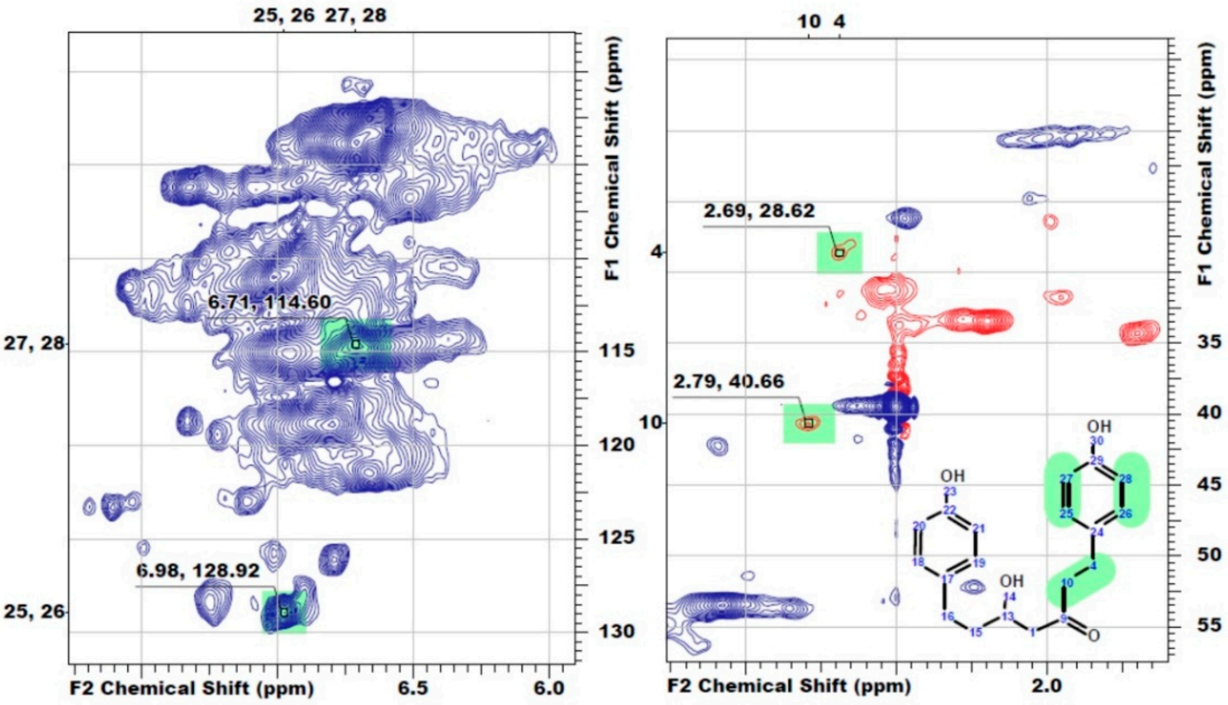

Figure S4. Comparison of the calculated ^{1}H-^{13}C HSQC spectrum of platyphyloside with the experimental spectrum for birch phloem.

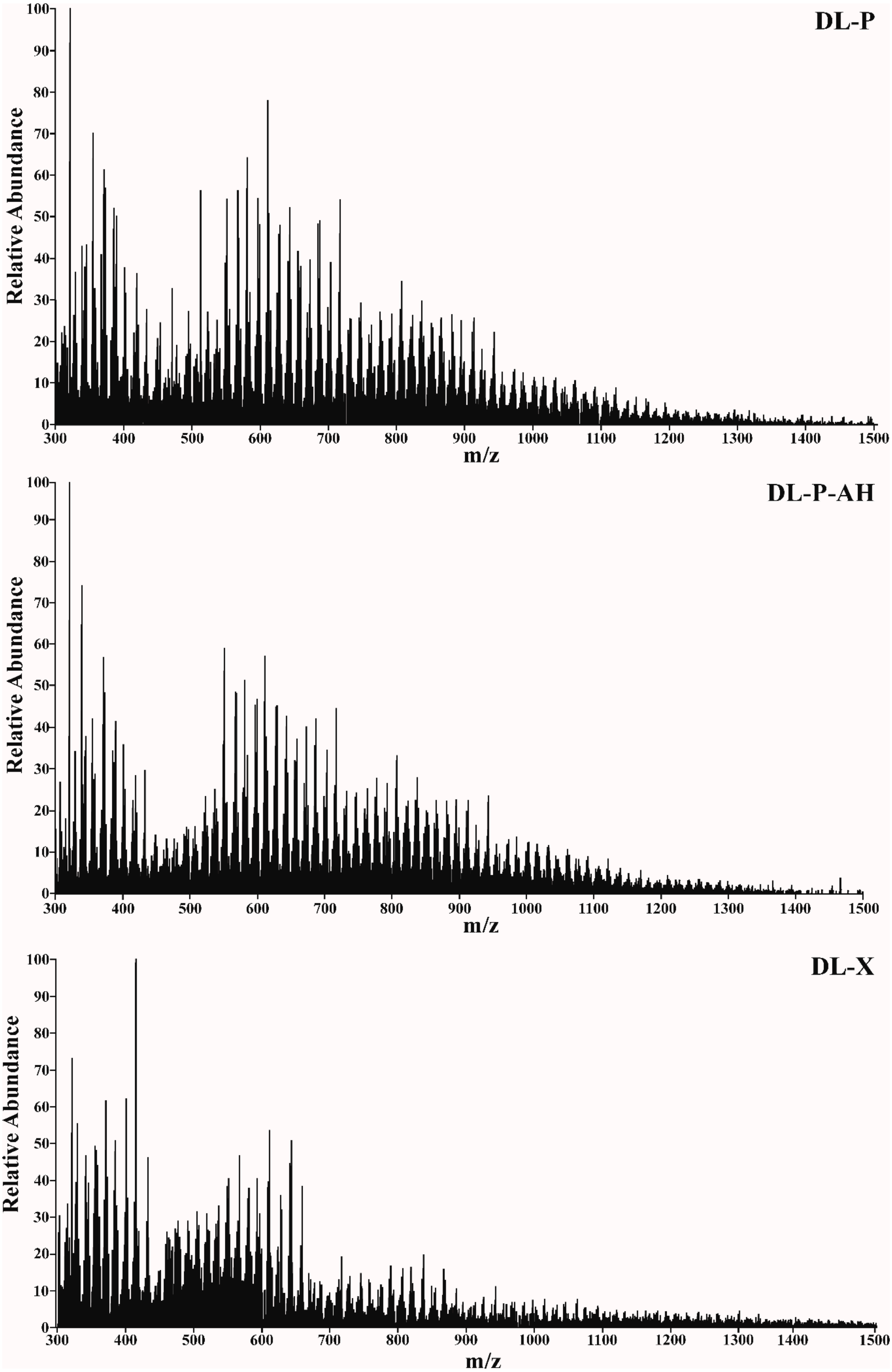

Figure S5. APPI-Orbitrap-HRMS mass spectra of DL-P, DL-P-AH and DL-X

Table S4. Relative intensities of balanofolin and methoxybalanofolin signals in birch xylem and phloem mass spectra

compound	$[M-H]^-$	*m/z*	Relative intensity, %		
			DL-X	DL-P	DL-P-AH
Balanopholin	$[C_{20}H_{19}O_6]^-$	355.1199	40	70	70
Methoxybalanopholin	$[C_{21}H_{21}O_7]^-$	385.1294	19	30	30

Table S5. Tandem mass spectra of precursor ion with *m/z* 301 in DL-P mass spectrum and quercetin analytical standard (collision energy 20 eV)

Product ion	m/z, Da	Relative intensity, %	
		Quercetin standard	DL-P
$[C_{15}H_9O_7]^-$	301.0354	35	40
$[C_{14}H_9O_6]^-$	273.0405	7	10
$[C_8H_3O_5]^-$	178.9986	45	39
$[C_7H_3O_4]^-$	151.0037	100	100
$[C_7H_5O_2]^-$	121.0295	18	31
$[C_6H_3O_2]^-$	107.0138	17	15
$[C_6H_5O]^-$	93.0343	3	5

Table S6. Major monomeric compounds tentatively identified in broadband CID tandem mass spectra of birch xylem and phloem lignin preparations (precursor ion m/z range 300–1000, collision energy 10 eV)

Assumed structures	$[M-H]^-$	*m/z*, Da	Structure type	Relative intensity, %		
				DL-X	DL-P	DL-P-AH
4-Hydroxybenzaldehyde	$[C_7H_5O_2]^-$	121.0284	H	8	7	6
2-Methoxyphenyl (guaiacol)	$[C_7H_7O_2]^-$	123.0441	G	8	8	7
4-Hydroxyacetophenone	$[C_8H_7O_2]^-$	135.0441	H	4	5	3
4-Hydroxybenzoic acid	$[C_7H_5O_3]^-$	137.0233	H	21	41	46
(p-Hydroxyphenyl)glyoxal	$[C_8H_5O_3]^-$	149.0244	H	15	22	21
4-Oxy-3-methoxybenzaldehyde (vanillin)	$[C_8H_7O_3]^-$	151.0401	G	35	28	30
p-Hydroxycinnamic acid	$[C_9H_7O_3]^-$	163.0400	H	10	9	13
4-Hydroxy-3-methoxybenzoic acid	$[C_8H_7O_4]^-$	167.0338	G	23	15	21
Coniferyl aldehyde	$[C_{10}H_9O_3]^-$	177.0557	G	12	8	11
(p-Hydroxy-3-methoxyphenyl)glyoxal	$[C_9H_7O_4]^-$	179.0352	G	25	19	21
2,4'-Dihydroxy-3'-methoxyacetophenone	$[C_9H_9O_4]^-$	181.0508	G	37	13	17
3-Methoxy-4-hydroxycinnamic acid	$[C_{10}H_9O_4]^-$	193.0507	G	39	26	34
3-methoxy-4-hydroxyphenylpyruvic acid	$[C_9H_7O_5]^-$	195.0300	G	12	5	7
3,5-Dimethoxy-4-hydroxyacetophenone	$[C_{10}H_{11}O_4]^-$	195.0664	S	15	12	11
Sinapaldehyde	$[C_{11}H_{11}O_4]^-$	207.0664	S	22	7	7
(p-Hydroxy-3,5-dimethoxyphenyl)glyoxal	$[C_{10}H_9O_5]^-$	209.0457	S	21	9	13
2,4'-Dihydroxy-3',5-dimethoxyacetophenone	$[C_{10}H_{11}O_5]^-$	211.0615	S	16	8	7
Sinapinic acid	$[C_{11}H_{11}O_5]^-$	223.0614	S	30	15	10
β-D-xylopyranose	$[C_5H_{10}O_5]^-$	149.0454	-	2	8	8
